# Cardio-postural interactions and muscle-pump baroreflex are severely impacted by 60-day bedrest immobilization

**DOI:** 10.1038/s41598-020-68962-8

**Published:** 2020-07-21

**Authors:** Da Xu, Malcom F. Tremblay, Ajay K. Verma, Kouhyar Tavakolian, Nandu Goswami, Andrew P. Blaber

**Affiliations:** 1 Department of Biomedical Physiology and Kinesiology, Simon Fraser University, Burnaby, BC V5A 1S6 Canada; 2 School of Electrical Engineering and Computer Science, University of North Dakota, Grand Forks, ND 58202 USA; 3 Physiology Division, Otto Loewi Research Center for Vascular Biology, Immunology and Inflammation, Medical University of Graz, Graz, Austria

**Keywords:** Systems biology, Dynamical systems, Cardiovascular biology

## Abstract

To understand fundamental mechanisms associated with post-flight orthostatic intolerance we investigated the interaction between the cardiovascular and postural functions before and after 60 days of head down bedrest (HDBR). Twenty healthy young males (35.0 ± 1.7 years) were subjected to 60-day HDBR at 6˚ to simulate spaceflight-induced fluid shifts. A supine-to-stand (STS) test was conducted to evaluate cardio-postural control before and after (R) HDBR while an assessment of cardiovascular function was performed during HDBR. Beat-to-beat heart period, systolic blood pressure, and electromyography impulses were derived for wavelet transform coherence and causality analyses of the cardio-postural control and used to assess changes in the muscle-pump baroreflex. During quiet stand of the STS test, compared to baseline, heart rate was 50% higher on the day of exit from bedrest (R0) and 20% higher eight days later (R8). There was a 50% increase in deoxygenated hemoglobin on R0 and R8. Leg muscle activity reduced, and postural sway increased after HDBR. Causality of the muscle-pump baroreflex was reduced on R0 (0.73 ± 0.2) compared to baseline (0.87 ± 0.2) with complete recovery by R8. The muscle-pump baroreflex also had decreased gain and fraction time active following HDBR. Overall, our data show a significantly impaired muscle-pump baroreflex following bedrest.

## Introduction

Spaceflight-induced weightlessness produces time-dependent physiological adaptation responses^1–3^. Once adapted to weightlessness, many responses become inappropriate upon return to a gravitational environment. The resulting physiological deconditioning could pose a serious challenge in the event of a critical post-landing situation requiring rapid escape from a spacecraft. Orthostatic intolerance after spaceflight remains a health and safety concern for astronauts, not just on landing day but also in the days of recovery^4^. Similar physiological deconditioning is seen following long lasting bedrest confinements (e.g. in young persons participating in head down bedrest studies, or in older persons due to chronic diseases or after falls and falls-related injuries)^5^.

Gravitational effects on the cardiovascular system during upright posture (orthostatic loading) induce a decrease in blood pressure which, if not compensated for, will cause loss of consciousness^6^. Baroreflex plays an essential role in maintaining blood pressure equilibrium under orthostatic loading. In regards to the arterial baroreflex, two processes exist in tandem: one in which blood pressure alters baroreceptor output and hence heart rate and vasomotor tone^7–9^; and, a second in which changes in heart rate and vasomotor tone alters blood pressure—the former a baroreflex (feed-back) and the latter a non-baroreflex (feed-forward) control of blood pressure^10^. Similarly, conscious movement could place a person in a position of instability, which would require post-movement adjustment to maintain an upright stance. Therefore, any change in body position (feed-forward) must be accompanied with an integrated correction to maintain balance. Any divergence from this path would require further feed-back response. With microgravity exposure affecting cardiovascular, sensory, and motor neuron activity, these cardiovascular and postural responses and associated spinal reflexes are altered, leading to post-flight dysfunctions^11^.

Novak et al.^12^ have shown the influence of skeletal muscle contractions during walking on blood pressure through feed-forward activity. Still others have suggested relationships between postural sway and blood pressure changes^13–15^. Over the past decade, based on such research, we have proposed the cardio-postural system as a new integrated approach to understanding cardiovascular regulation in relation to postural control^16–20^. In 2012, we presented a detailed description of this model and postulated on the impact of spaceflight on its cardiovascular, sensory motor, postural and skeletal muscle components and their interactions^11^. A major tenant of the model is that the baroreflex can activate skeletal muscle in the lower legs via the postural control system (i.e., muscle-pump baroreflex). Upon standing, decreased blood pressure activates reflex responses, through a hypothesized cardio-postural control center, including elevation of heart rate, vascular resistance (via arterial baroreflex) and skeletal muscle pump activity (via muscle-pump baroreflex). The muscle-pump baroreflex responds to blood pressure changes and activates skeletal muscle contractions to combat blood pooling during standing and maintain blood pressure. Our research has shown that the muscle-pump baroreflex plays an important role in orthostasis^17–20^.

Prolonged head down bedrest (HDBR), characterized by immobilization, inactivity, confinement, and reduced pull of gravity, is an effective model to simulate physiological changes in humans when exposed to microgravity^5,21^. Similar adaptations of physiological systems during spaceflight have also been observed during and after HDBR^22–26^. In addition, prolonged HDBR has been shown to have adverse effects on both the vestibular system^27,28^ and postural stability^29^. With limited resources for human research during spaceflight, prolonged HDBR provides an ideal experimental setting to study post-flight deconditioning in astronauts. A comprehensive review on HDBR as a simulation model of spaceflight has been conducted by Pavy-Le Traon et al.^30^.

With cardiovascular control coupled to the postural skeletal muscle system, the significance of spaceflight deficits in both systems become compounded post-flight. The purpose of this study was to further our understanding of fundamental adaptive homeostatic mechanisms associated with post-flight orthostatic intolerance (OI) on cardiovascular and postural functions. In this study, we investigated the cardio-postural system in terms of the muscle-pump baroreflex before and after prolonged (60 days) HDBR as an analogue for the microgravity during spaceflight. We have developed a series of techniques to capture and segregate the cardiovascular and postural components associated with standing. Wavelet transform coherence (WTC) analysis^17,18,20^ and convergent cross mapping (CCM) causality^19,31,32^ methods have been adapted to extract indices characterizing the interaction time (*fraction time active, FTA*), response gain value (*gain*), and control directionality (*causality*) among cardiovascular and postural muscle measurements. Based on our previous evidence of an interactive system regulating blood pressure through cardio-postural interactions^18–20^, in this study we hypothesized that: (1) *the cardio-postural interaction, in terms of FTA, gain, and causality, is reduced and associated with cardiovascular and postural deconditioning after HDBR*; (2) *the cardio-postural impairment is reflex/neurally mediated and not due to changes in muscle-pump mechanics.*

## Results

### Participants

Twenty male volunteers were entered into the study with ten participants randomly assigned to nutritional supplementation treatment group (intervention) and ten to placebo group (control). Data from 19 participants (age: 35.0 ± 1.7 years, height: 1.76 ± 0.01 m, body mass: 72.9 ± 1.7 kg; mean ± SEM) were used for analysis with one participant in the control group excluded due to non-compliance with bedrest and study rules (described in Supplemental Materials).

### Body mass

On the first day after bedrest their body mass was on average 0.7 kg lighter (72.2 ± 1.6 kg, p < 0.01) than when they entered the study. This was recovered by the end of the study (R8: 73.1 ± 1.6 kg).

### Othostatic tolerance

The orthostatic tolerance time was not affected by the cocktail intervention (F_(1,17)_ = 0.30, p = 0.59), and there was no interaction between Cocktail and HDBR (F_(1,17)_ = 0.01, p = 0.92); however, it was reduced follow HDBR. The time to presyncope changed from 24.3 ± 5.7 min on BDC02 to 11.3 ± 6.6 min on R0 (F_(1,17)_ = 63.02, p < 0.0001).

### Supine and HDBR cardiovascular measures

**Figure 1:**
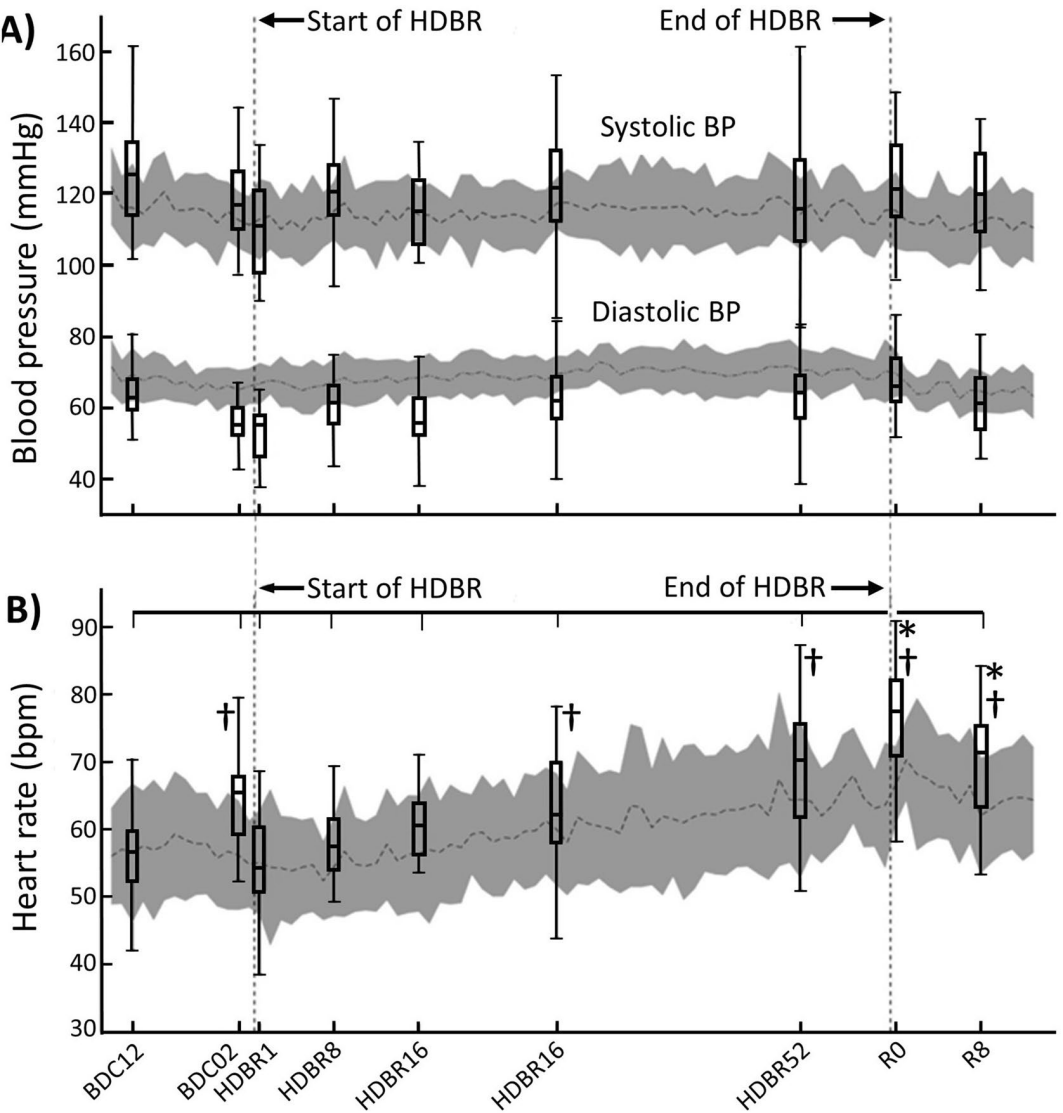
Blood pressure (**A**) and heart rate (**B**) measurements during supine (pre- and post-HDBR) and HDBR. Dashed horizontal lines with shaded areas represent the mean ± s.d. of the daily measurements by MEDES staff at 7:30 am one half hour after waking and before breakfast. The box plots represent the mean (25th–50th percentile) ± range of our measurements made between 10 and 11 am in supine pre-, post-HDBR, and during HDBR. The vertical lines projecting from the horizontal line above (**B**) represent the supine data collected by our group that were significantly different from R0. ^**†**^Data that were different from BDC12. *Data that were different from BDC02.

There was no effect of Cocktail on supine or HDBR SBP (F_(1,17)_ = 0.026, p = 0.874), and there was no interaction between Cocktail and Test-day (F_(8,136)_ = 0.654, p = 0.752); therefore, these data were collapsed to a one-way ANOVA for analysis. No changes in supine or HDBR resting SBP were observed (Fig. 1). As a check, we compared our measured values for systolic and diastolic blood pressure with that of osculation performed daily by the MEDES medical staff during morning check-up at 7:30 am one half hour after waking and before breakfast using an automated device, Dynamap Pro 300 (GE) (Fig. 1). Although variability in measurements occurred, none was significantly different from the medical data collected 2 h earlier.

Neither supine nor HDBR HR was altered by Cocktail intervention (F_(1,17)_ = 0.032., p = 0.860), and there was no interaction between Cocktail and Test-day (F_(8,136)_ = 0.748, p = 0.649). As a consequence, these data were collapsed to a one-way ANOVA for analysis. Supine or HDBR resting HR was significantly increased from beginning to end of HDBR (Fig. 1, F_(8,136)_ = 37.71, p < 0.0001). On the first day of HDBR (HDBR1), HR was 55 ± 2 bpm. By HDBR16 it was significantly higher (61 ± 2 bpm) and by HDBR52, HR was significantly higher than HDBR16 at 69 ± 2 bpm. Pre-HDBR there was a significant difference between baseline HR on BDC12 (56 ± 2 bpm) and HR on BDC02 (64 ± 2 bpm), while post-HDBR both our measured supine HR values (R0: 76 ± 2 bpm; R8: 70 ± 2 bpm) were significantly higher than both BDC12 and BDC02 (Fig. 1).

### Effects of 60-day HDBR on standing cardiovascular and postural measures

The cardiovascular and postural systems were significantly affected by 60-day HDBR as indicated by the large number of measured variables showing significant changes with HDBR and over Test-days (Table 1).

**Table 1:** Results of three-way repeated measures Analysis of Variance (Cocktail, head-down bedrest and test day) of standing values from the supine-to-stand test.

Variable	Cocktail		HDBR		Test day(HDBR)		HDBR × cocktail		Test day × cocktail (HDBR)	
	F_(1,17)_	p	F_(1,51)_	p	F_(2,51)_	p	F_(1,51)_	p	F_(2,51)_	p
Cardio-postural variables										
HR	0.009	0.924	142.9	** < 0.001**	47.14	** < 0.001**	0.271	0.605	0.440	0.645
SBP	0.765	0.384	6.244	**0.016**	2.744	0.074	0.044	0.835	3.118	0.053
EMG	0.727	0.406	4.498	**0.039**	1.465	0.241	0.001	0.979	0.788	0.460
EMGimp	0.621	0.442	26.21	** < 0.0001**	0.425	0.656	0.001	0.978	0.080	0.455
COPr	2.16	0.16	41.46	** < 0.0001**	12.22	** < 0.0001**	2.788	0.101	2.183	0.123
COPr_v_	0.072	0.792	77.64	** < 0.0001**	23.54	** < 0.0001**	1.714	0.196	1.176	0.317
∆deoxy Hb	0.002	0.964	15.13	**0.0003**	3.447	**0.040**	0.966	0.330	0.382	0.685
∆Oxy Hb	0.003	0.958	0.134	0.717	0.144	0.866	0.162	0.670	0.792	0.459
Blood pressure variability										
A_HF_	0.472	0.503	0.686	0.414	0.235	0.791	2.375	0.130	2.618	0.083
A_LF_	2.697	0.119	8.951	**0.004**	1.916	0.158	0.031	0.861	0.982	0.382
LF/HF	2.012	0.174	2.891	0.095	2.529	0.090	1.508	0.225	0.368	0.694
Muscle-pump baroreflex										
Fraction time active										
HF	0.219	0.646	6.791	**0.012**	6.785	0.111	1.223	0.273	0.978	0.383
LF	0.015	0.09	21.68	** < 0.0001**	11.11	** < 0.0001**	0.170	0.682	1.056	0.355
VLF	1.910	0.185	3.208	0.079	2.132	0.129	0.049	0.824	2.326	0.108
Gain										
HF	0.356	0.559	0.416	0.522	2.113	0.129	1.381	0.356	0.803	0.454
LF	0.032	0.861	8.007	**0.0066**	0.744	0.5	0.224	0.638	0.615	0.544
VLF	0.966	0.339	15.66	**0.0002**	0.074	0.929	0.603	0.441	0.617	0.544
Causality										
SBP → EMGimp	0.547	0.47	29.25	** < 0.001**	5.276	**0.008**	0.908	0.345	1.323	0.275
EMGimp → SBP	0.023	0.881	0.112	0.74	0.205	0.816	0.481	0.491	1.718	0.190

HDBR: head-down bedrest; HR: heart rate; SBP: systolic blood pressure; EMG: electromyogram; EMG_imp_: Electromyogram beat-to-beat impulse; COPr: mean deviation from the center of pressure; COPr_v_: mean velocity of movement of COPr; ∆deoxyHb: change from initial value at the beginning of stand in deoxygenated hemoglobin in the medial gastrocnemius; ∆OxyHb: change from initial value at the beginning of stand in oxygenated hemoglobin in the medial gastrocnemius. Blood pressure variability were determined from the final 5 min of stand; A_HF_: square root of High Frequency power, A_LF_: square root of Low Frequency power; LF/HF: low frequency to high frequency ratio.

Bolded values highlight results where p < 0.05.

#### Cardiovascular

All cardiovascular and postural measures except oxygenated Hb were affected by HDBR (Table 1). During the quiet stand of the STS test 90 min after the end of bedrest on R0, the average HR was 50% higher, and on R8 20% higher, compared to baseline on BDC12. There was a 50% increase in deoxygenated hemoglobin from the beginning to the end of standing on R0 compared to BDC12 with a similar result on R8. No changes in the oxygenated hemoglobin response during standing were observed on R0 or R8. Standing SBP on R0 was also lower than baseline (BDC12) (Table 2). Although there was not significant main effect for Cocktail, there was as a close to significant interaction with the Cocktail and Test-day (p = 0.053, Table 1). Post-hoc analysis indicated that standing SBP for cocktail intervention participants was lower on R0 (116 ± 6 mmHg) compared to BDC12 (138 ± 6). No other comparisons with or between control and cocktail intervention were found to be significant.

**Table 2:** Mean standing cardio-postural values and blood pressure variability.

	HR (bpm)	SBP (mmHg)	EMG (µV)	EMG_imp_ (µV·s)	COPr (mm)	COPr_v_ (mm/s)	∆deoxy Hb (V)	∆Oxy Hb (V)	BPV		
									A_HF_ (mmHg)	A_LF_ (mmHg)	LF/HF
BDC12	84 ± 3	138 ± 4	82.7 ± 7.7	60.2 ± 5.6	3.8 ± 0.4	8.0 ± 1.0	4.4 ± 0.5	− 2.1 ± 0.5	2.73 ± 0.32	4.34 ± 0.41	2.21 ± 0.26
BDC02	82 ± 3	129 ± 4	86.8 ± 7.7	64.6 ± 5.6	3.8 ± 0.4	7.5 ± 1.0	5.3 ± 0.5	− 2.2 ± 0.5	2.90 ± 0.32	5.08 ± 0.41	1.98 ± 0.26
R0	129 ± 3*^†^	123 ± 4*	**79.2 ± 7.7**	**38.5 ± 5.6**	6.9 ± 0.4*^†^	17.8 ± 1.0*^†^	7.0 ± 0.5*^†^	− 2.2 ± 0.5	2.64 ± 0.32	**5.89 ± 0.41**	2.38 ± 0.26
R8	96 ± 3*	**128 ± 4**	**64.4 ± 7.7**	**41.9 ± 5.6**	**4.7 ± 0.4**	**10.7 ± 1.0**	6.5 ± 0.5*	− 2.2 ± 0.5	2.70 ± 0.32	**5.42 ± 0.41**	2.46 ± 0.26

Mean cardio-postural values were obtained from the final 5-min of stand. HR: heart rate; SBP: systolic blood pressure; EMG: electromyogram; EMG_imp_: Electromyogram beat-to-beat impulse; COPr: mean deviation from the center of pressure; COPr_v_: mean velocity of movement of COPr; ∆deoxyHb: change from initial value at the beginning of stand in deoxygenated hemoglobin in the medial gastrocnemius; ∆OxyHb: change from initial value at the beginning of stand in oxygenated hemoglobin in the medial gastrocnemius; BPV: blood pressure variability was determined from the final 5 min of stand; A_HF_: square root of High Frequency power, A_LF_: square root of Low Frequency power; LF/HF: low frequency to high frequency ratio. BDC12: baseline data collection day − 12; BDC02: baseline data collection day − 02; R0, recovery day 0; R8: recovery day + 8. Bolded region: indicates there was a significant difference between pre- and post-HDBR. If there was a significant Test-day × HDBR interaction, then the following tests of significance are presented: *: different from BDC12 (p < 0.05); ^**†**^: different from BDC02 (p < 0.05).

#### Electromyography and posturography

Overall lower leg muscle activity was reduced with HDBR (Tables 1, 2), with a trend to lowest values on R8 (p = 0.06). When EMG was integrated beat-to-beat (EMG_imp_), the effect was more dramatic, with more than a 35% reduction from baseline on R0 and a similar result on R8. Postural sway was increased following HDBR, with an increased mean deviation from the center of pressure (COPr) and mean sway velocity (COPr_v_) which more than doubled on R0 (Table 2).

#### Blood pressure variability (BPV)

No changes in BPV were observed as a function of Cocktail, but there was an increase in LF power amplitude with HDBR (Table 1, 2).

#### Muscle-pump baroreflex

In the muscle-pump baroreflex direction, where the skeletal muscle responses to changes in blood pressure through baroreflex, a significant reduction in gain was seen with HDBR in LF and VLF regions (Tables 1, 3). In both cases, there was a trend to reduced gain immediately after HDBR (R0) with a further significant decline by R8. This pattern of change in gain was reflected in the FTA index between SBP and EMG_imp_. In HF and LF bands, FTA was reduced post-HDBR with a significant reduction in LF FTA observed on R0 and not on R8 (Tables 1, 3).

**Table 3:** Wavelet transform analysis of systolic blood pressure and calf muscle electromyography impulse interactions during standing.

	SBP → EMG_imp_							EMG_imp_ → SBP
	Gain (HF) (µV·s/mmHg)	FTA (HF)	gain (LF) (µV·s /mmHg)	FTA (LF)	gain (VLF) (µV·s /mmHg)	FTA (VLF)	Causality	Causality
BDC12	1.54 ± 0.21	0.22 ± 0.03	0.72 ± 0.10	0.52 ± 0.06	0.93 ± 0.09	0.24 ± 0.04	0.87 ± 0.02	0.92 ± 0.01
BDC02	1.42 ± 0.21	0.21 ± 0.03	0.87 ± 0.10	0.52 ± 0.06	0.93 ± 0.09	0.27 ± 0.04	0.88 ± 0.02	0.91 ± 0.01
R0	1.59 ± 0.21	**0.12 ± 0.03**	**0.55 ± 0.10**	0.20 ± 0.06*^†^	**0.61 ± 0.09**	0.17 ± 0.04	0.73 ± 0.02*^†^	0.91 ± 0.01
R8	1.09 ± 0.21	**0.19 ± 0.03**	**0.47 ± 0.10**	**0.47 ± 0.06**	**0.55 ± 0.09**	0.24 ± 0.04	**0.82 ± 0.02**	0.91 ± 0.01

Systolic blood pressure (SBP), muscle electromyography impulse (EMG_imp_), Wavelet transform gain (gain), Fraction Time Active (FTA) in the High Frequency (HF), Low Frequency (LF) and Very Low Frequency (VLF) bands. BDC12: baseline data collection day − 12; BDC02: baseline data collection day − 02; R0, recovery day 0; R8: recovery day + 8. Bolded region: indicates there was a significant difference between pre- and post-HDBR. If there was a significant Test-day × HDBR interaction, then the following tests of significance are presented: *: different from BDC12 (p < 0.05); ^**†**^: different from BDC02 (p < 0.05).

#### Causality

The CCM assessment of SBP-EMG_imp_ directional coupling showed a significant reduction in the causality in the baroreflex direction (SBP → EMG_imp_) from 0.87 ± 0.02 on BDC12 to 0.73 ± 0.02 on R0 with complete recovery on R8. No change in causality was observed in the opposite direction (EMG_imp_ → SBP) with a value constantly at 0.91 (Table 3).

#### Active gain vs causality

**Figure 2:**
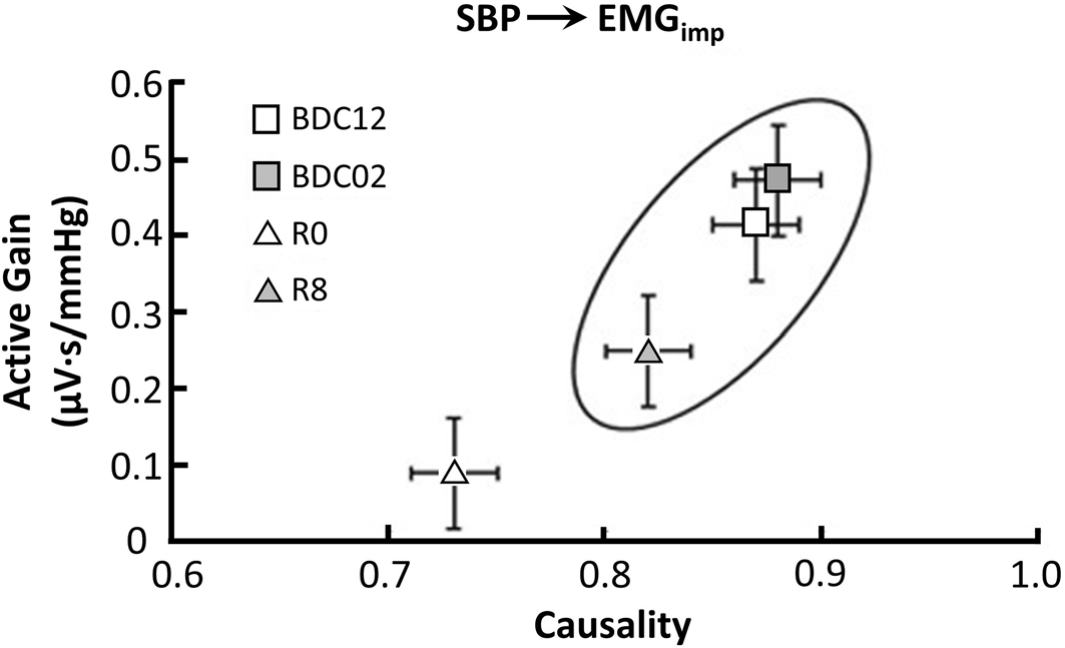
The interrelationship between causality and low frequency skeletal muscle-pump baroreflex gain as a function of active interaction time (Active Gain: SBP → EMG_imp_ gain X fraction time active) on all four supine-to-stand test days. Circled data are not significantly different.

When muscle-pump active gain was plotted as a function of causality, R0 was observed to be isolated in the lower left quadrant (Fig. 2); a region of low active gain and causality. On R8, the plot clearly shows the transition of control back in the baroreflex direction (high causality), yet the activity and gain of the reflex have not returned to pre-HDBR values. On R8, the system was found to be not significantly different from pre-HDBR, however the muscle-pump baroreflex gain remained significantly lower on R8 while causality had only partially returned (Table 3).

### Calf physical measurements

Measurements of calf circumference showed a significant reduction from pre-HDBR (36.5 ± 0.5 cm) to R0 (34.1 ± 0.5 cm, p < 0.01). Calf circumference returned to pre-HDBR values by R8 (36.1 ± 0.5 cm). Skin fold thickness increase from pre-HDBR (9.6 ± 1.0 mm) to R0 (11.3 ± 1.0 mm, p < 0.001) without recovery by R8 (11.1 ± 1.0 mm).

## Discussion

**Figure 3:**
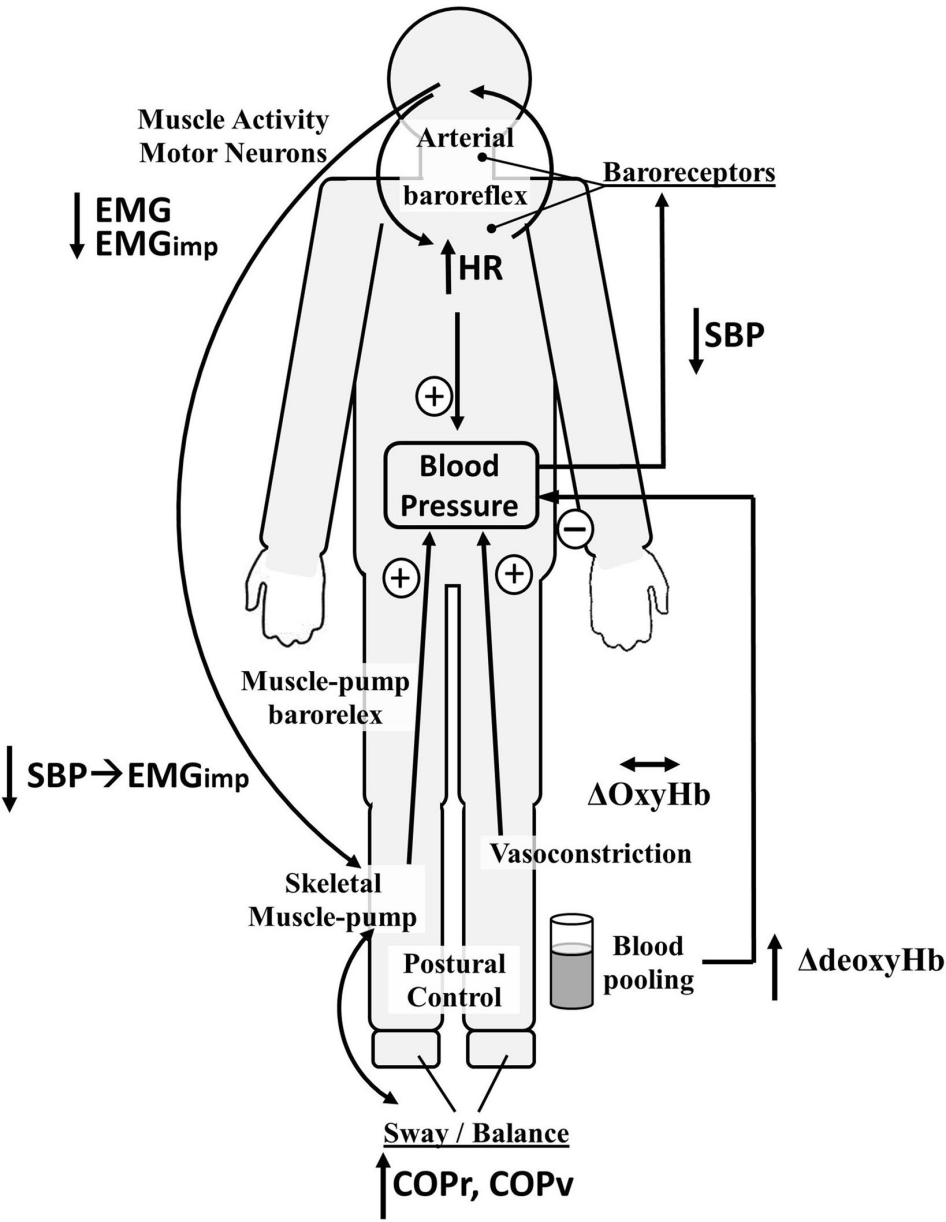
The cardio-postural system and the effects of bedrest. Upon standing, blood pools in the lower legs and the resultant drop in blood pressure is detected by baroreceptors to relay this information to the brain where: (1) the arterial baroreflex increases heart rate and vascular resistance; and (2) the skeletal muscles in the legs contract (skeletal muscle-pump), propelling pooled blood in the leg veins back to the heart (muscle-pump baroreflex). Our data show that 60 days of 6˚ head-down tilt bedrest produces in large changes in the control of blood pressure and skeletal muscle in the lower limbs. Overall reductions in systolic blood pressure (SBP), muscle electromyography (EMG, and beat-to-beat muscle EMG impulse) and increases in heart rate (HR), venous blood pooling (∆deoxyHb), and postural sway (COPr, COPv) were seen during standing immediately following bedrest. Reduced gain, causality and fraction of time active for muscle-pump baroreflex (SBP → EMG_imp_) were also observed. (↓, decrease; ↔ , no change; ↑, increase).

Our novel data obtained from prolonged bedrest immobilization show that following bedrest, skeletal muscle activation in relation to blood pressure regulation is significantly impaired. To our knowledge, this is the first study that has shown changes of cardio-postural interactions following prolonged bedrest confinement (Fig. 3). The results obtained are particularly important for understanding OI, a condition that frequently occurs in older persons^5^ or younger persons following bedrest confinement or in simulated microgravity (e.g. HDBR)^33^, or in astronauts post-spaceflight^4,34^ respectively.

Elevated HR over the course of the HDBR (Fig. 1) and during standing after HDBR was an indication of cardiovascular deconditioning, which persisted up until R8 when we made our final measurements (Table 2). This was a response to increased venous pooling in the lower limbs, as indicated by the persistent higher deoxygenated Hb measurements^35^, and the lack of increased vasoconstriction in the same region, as shown by the lack of change in oxygenated Hb (Tables 1, 2). The mean standing SBP was only slightly decreased on R0; however, this decrease in standing SBP was observed in the nutritional intervention group, not the control group. This could have been most likely a result of the blood pressure lowering effect of selenium^36^, vitamin E^37^, and omega-3-acid ethyl ester^38^.

Postural stability was impaired with both an increase in resultant COP (COPr, i.e., deviation from upright stance) and the average velocity of sway (COPr_v_) (Table 2). This may have been due to the reduction in overall activity of the leg muscles (EMG) during standing. Any perturbation in body motion would have required more time for correction. The EMG impulse, which we have used as an expression of muscle activity input in relation to the skeletal muscle-pump, had an even greater decrease from pre-HDBR values on R8 (Table 2). This was a strong indication that the ability of the skeletal muscle pump in response to blood pressure changes was compromised.

Following HDBR there was a significant reduction in the capacity of the skeletal muscle-pump to respond to changes in BP. Of the three frequency bands, LF presented the highest percentage of engagement between SBP and EMG_imp_, indicating that this frequency band may represent the most important rhythms in terms of BP regulation within the 5-min stand. The reduction in FTA in the LF band was also the greatest with an absolute reduction of 30% from a baseline of 52% (Table 3). These data indicate that 60 days of HDBR resulted in a reduced engagement of the baroreflex system control of skeletal muscle, which may be a component of the decreased standing beat-to-beat EMG impulse observed after HDBR (Table 2). This is supported by the lack of change in the vasoconstrictor estimate (OxyHb, Table 2) with respect to vascular SNS activity.

**Figure 4:**
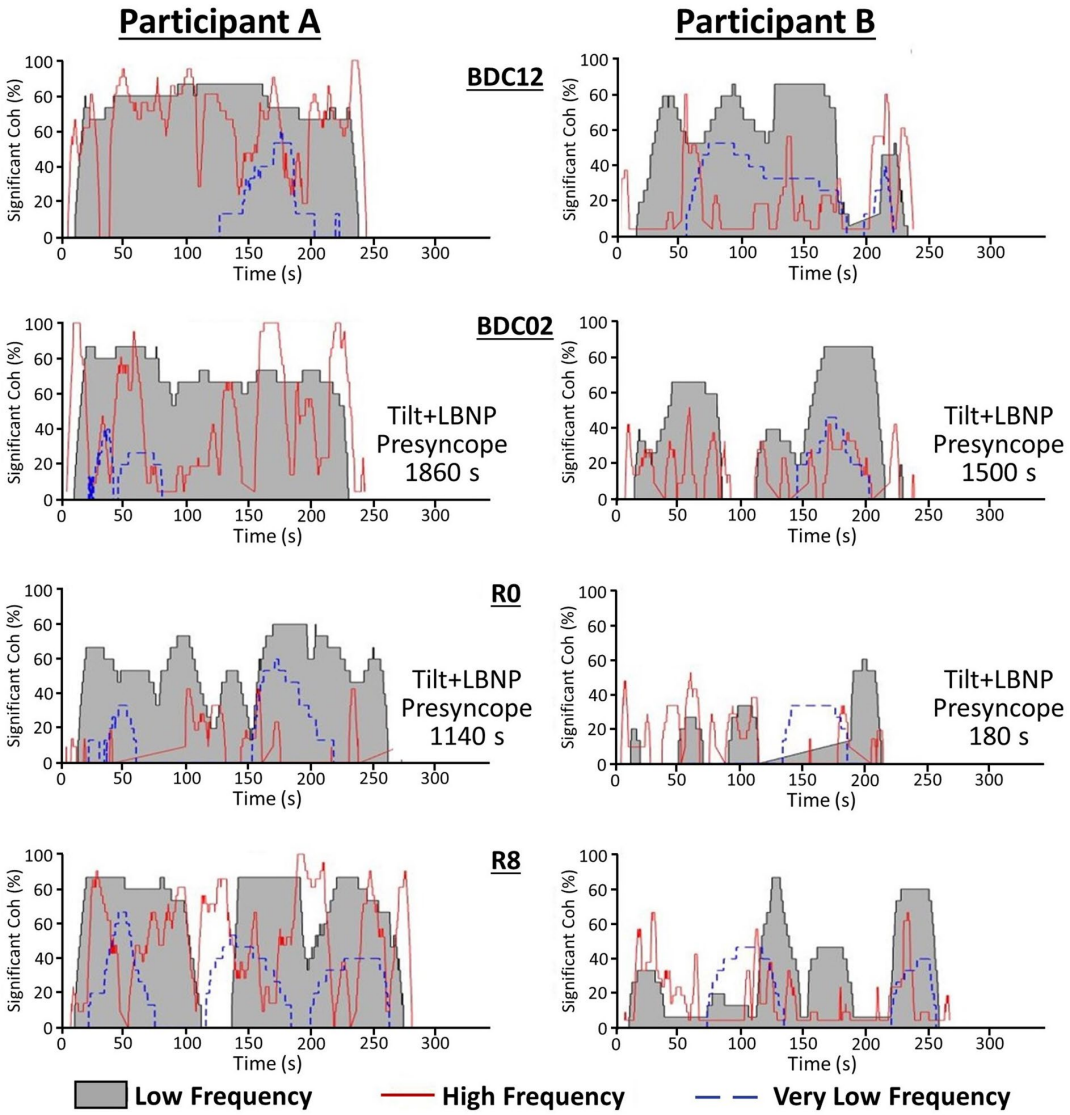
Percent significant coherence—time map of SBP → EMG_imp_ for a participant with high (**A**) and a participant with low (**B**) orthostatic tolerance immediately after bedrest (R0). Significant Coh (%): percentage of the frequency bands in which coherence was above the significance threshold. BDC12: baseline data collection day − 12; BDC02: baseline data collection day − 02; R0, recovery day 0; R8: recovery day + 8. TILT + LBNP: head-up tilt followed by lower body negative pressure until presyncope (see “Methods”).

The altered interaction of BP to muscle activation (gain, FTA, causality) on R0 and R8 suggests not only a possible decrease in the reflex output to the muscle but also a change in activation. These can be visually presented by plotting the percentage of significant coherence over the duration of the stand, as is used to determine FTA for a given participant. For purposes of discussion we have chosen two participants at either end of the post-HDBR orthostatic tolerance spectrum (Fig. 4).

The LF band which was found to have the greatest engagement of the skeletal muscle-pump baroreflex (Table 3) is represented by the grey zone. At each time point, the coherence was evaluated based on the threshold of significant coherence obtained from a Monte Carlo simulation^20^. In each frequency band (HF, LF, and VLF), the number of frequency sub-band segments with significant coherence was calculated. At each time point, if two or more frequency sub-band segments in a frequency band had significant coherence, the value was plotted as a percentage of the total number of frequency sub-band segments in that frequency band (Fig. 4). The participant on the left only presented a moderate loss of orthostatic tolerance over the 60-day bedrest. The participant on the right had a large decrease in orthostatic tolerance (Fig. 4).

Our data of FTA (Table 3), indicated a significant reduction in muscle-pump baroreflex on R0. This is evident in these graphs with a reduction in the grey area on R0, particularly in the participant with lower orthostatic tolerance. After HDBR both participants exhibited a visually more pulsatile EMG interaction with SBP, which was more pronounced in the participant with lower orthostatic tolerance. In fact, this participant exhibited these characteristics pre-HDBR, suggesting a less active or interactive muscle-pump baroreflex. The participant with higher tolerance did appear to have an increase in the pulsatile pattern partially observed on BDC02 and exaggerated on R0 and R8.

The reduction in coupling in the muscle-pump baroreflex direction (SBP → EMG_imp_) was supported by the reduced causality at R0 where there was a similar reduction of FTA in the LF band (Table 3). Impaired arterial baroreflex was also reported after bedrest^23,39–41^. The lack of causality changes in the inverse direction (EMG_imp_ → SBP) indicates that the mechanical coupling from muscle-pump activity to BP was unaltered by HDBR. This gives further support to the hypothesis that changes in BP regulation are reflex/ neurally mediated and not due to changes in mechanical muscle-pump mechanics.

In this study we have reported an impaired muscle-pump baroreflex after HDBR. Reductions in the cardiac arterial baroreflex response has been long reported for both short-term^42–44^ and long-term^45^ spaceflights. By showing the existence of reductions in the reflex mediated muscle-pump response to BP changes following HDBR, we hypothesize similar declines in the muscle-pump baroreflex after spaceflight of comparable duration (~ 2 months). Our results therefore provide unique ground-based reference data on changes in post-flight orthostatic BP regulation. The effects of flight duration on the degree of impairment of the baroreflex system require further investigations.

Since many negative effects of living in space parallel those of the aging process, such as OI, osteoporosis and muscle loss, our results have application in geriatrics, especially as older persons often feel dizzy upon standing up^5^. Function and coordination of somatosensory, vestibular, and postural systems for maintaining standing balance have long been noted to deteriorate with increasing age^46–48^. Impairment of cardiovascular regulatory functions with aging is considered as another risk factor of falls^49,50^. Based on the observation that both postural and BP control deficiencies are prevalent in normal aging, it is expected that the cardio-postural model could provide a more comprehensive way to describe the BP regulation mechanism in older persons. We have shown reductions in muscle-pump baroreflex in older compared to young persons^51^, which is in agreement with the results of the current study.

## Limitations and future work

This study was carried out in healthy young subjects. To what extent the results of this study are extendable to older persons—many of whom are on multiple medications and who have significant amount of sarcopenia even prior to bedrest confinement^5,52^. Needs to be investigated. Furthermore, older persons are confined routinely to bedrest either due to acute infections, traumatic injuries, operations or chronic diseases. Future studies should examine how cardio-postural interactions are influenced by varying periods of bedrest confinement in older patients. This is important as falls and fall related injuries commonly occur upon change of posture (from supine to standing or from sitting to standing position)^5,53,54^. In addition, only male participants were included in this study while women are even more prone to OI in the age ranges found in this study^6,55–59^.

Future studies should assess the effect of nutrition, together with exercise, on muscle and hemodynamic parameters during bedrest immobilization. This is important as physical activity has been singly identified as an important intervention in preventing deleterious effects of bedrest confinement^60,61^. Additional interventions that could be tested include cognitive training^21^, LBNP^62^, and artificial gravity^63^; all of these have been shown to alleviate the symptoms of bedrest induced physiological deconditioning.

The cardio-postural model introduced in this paper did not include other variables that could affect postural responses such as visual (eye-closed during tests) and vestibular inputs. A more comprehensive model incorporating the above variables should be adapted and investigated in future studies.

## Methods

### Study design and testing protocols

The experiments were conducted as part of a ESA funded prolonged HDBR study at the *Institut de Médecine et de Physiologie Spatiales* (MEDES), a *Centre National d’Études Spatiales* (CNES) operated facility in Toulouse, France. Each of the two HDBR campaigns lasted for 60 days and required participation of 10 volunteers subjected to HDBR at 6 degrees to simulate spaceflight induced fluid shifts. Ethical approval for all research was obtained from the *Comité de Protection des Personnes / CPP Sud-Ouest Outre-Mer I* and the *Agence Française de Sécurité Sanitaire des Produits de Santé* for each aspect of the study and scientific protocols. Research associated with our study was approved by the Office of Research Ethics at Simon Fraser University. The participants signed a written informed consent and were required to be available at MEDES for the entire 3-month study period. Research was conducted in in compliance with the guidelines and regulations of the above agencies.

The selection criteria and the number of participants required to show statistical significance were based on previously published work in this area^64–69^ and were agreed upon by MEDES and the 16 scientific teams involved. The scientific teams coordinated their activities and schedules with MEDES staff to ensure there was no overlap to minimize interference between teams’ measurement protocols as well as with ESA bedrest core data collection. Detailed inclusion and exclusion criteria are available in the section “Supplemental Material”. Bedrest studies with various duration ranging from several hours to several months have been performed^21,70,71^. As a balance between the attempt to simulate the effects of long-term spaceflight and a reasonable period for healthy volunteers to spend in bed, the present study consisted of 60-day 6 degree HDBR which was adopted by many other bedrest studies^26,72,73^.

In this bedrest campaign, the experimental condition (Cocktail) was a nutritional supplementation treatment. Participants were divided randomly into two groups with either a nutritional supplementation (intervention) or placebo (control) condition. Half of the participants received daily capsule supplements of antioxidants, vitamin E-selenium, and omega-3, which are described in detail in “Supplemental Material”. It was hypothesized that the nutritional condition would not affect the cardio-postural system.

Included in the MEDES protocol was a head-up tilt (HUT) + graded Lower Body Negative Pressure (LBNP) sequence to pre-syncope, a standard measure of orthostatic tolerance^74–76^. This HUT-LBNP sequence consisted of a 5-min supine baseline followed by an 80˚ head-up tilt for 15 min. Lower body negative pressure was then added in − 10 mmHg increments every 3 min until presyncope. The results of the orthostatic tolerance test are reported as the total time from start to presyncope. This ESA mandated test was prioritized to occur 2 days prior to bedrest and immediately upon completion of bedrest. To observe the temporal effects of recovery, we scheduled our baseline test prior to the first HUT-LBNP test on BDC12 to avoid interactions related to presyncope prior to testing. A follow-up test 45 min after the HUT-LBNP test on BDC02 to provide a basis of comparison with R0 since our post-HDBR measures were scheduled to follow the post-HDBR orthostatic tolerance testing at a similar time. There was no HUT-LBNP prior to our final test on R8, which provided a comparison to BDC12. It was carried out prior to our second supine-to-stand (STS) data collection in baseline and on the first day after exit from bedrest (see details of our protocol below).

**Figure 5:**
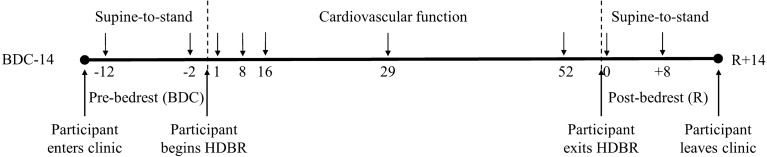
Timeline—the participants remained at the testing facility for a total of 76 days, of which 60 days were spent in 6˚ head-down tilt bedrest (HDBR). Participants arrived at MEDES 14 days prior to entering HDBR. At this time baseline data collection (BDC) was performed. After bedrest, participants remained at the clinic for 14 days where recovery (R) data were collected.

Two testing protocols were implemented depending on the various phases of the study. Before (BDC) and after (R) the HDBR experiment, the STS test was conducted to evaluate cardio-postural control. During the supine portion of the STS test and during HDBR an assessment of cardiovascular function was performed. For comparison, the supine portion of the STS and the HDBR analyses were standardized by using only the final 5 min of data in each. An STS test was conducted at the same time each morning 12 days (BDC12) and 2 days (BDC02) before HDBR, and on the day of (R0) and 8 days after (R8) exit from HDBR. The STS tests performed on BDC02 and R0 were conducted 45 min after the ESA bedrest core data syncope test. HDBR cardiovascular function tests were conducted at the same time each morning on HDBR days 1, 8, 16, 29 and 52 (Fig. 5).

#### Supine-to-stand

A supine-to-stand (STS) test was used to evoke the cardio-postural control system^16–20,77^. Upon arrival at the testing room, the participants were placed in the supine position and instrumented for blood pressure (BP), electrocardiography (ECG), lower leg electromyography (EMG), and near-infrared spectroscopy (NIRS) of the left medial gastrocnemius. Following instrumentation, which took 20 min, continuous data acquisition was started with the room lights turned off and 5-min eyes closed quiet supine data collected. This was followed by a 6-min quiet stand. The participants were asked to open their eyes and be assisted to the standing position. One experimenter would sweep their legs off the bed and another would raise their torso. Together, they would position the participant on the center of a force platform. Participants’ feet were placed parallel and 5 cm apart on the center of the force platform. During the 6-min quiet stand, they were instructed to keep their eyes closed with their arms relaxed at their sides, maintain imaginary eye-level gaze, and not to alter foot placement. Participants were instructed to close eyes during data collection because postural sway increases with the removal of visual input, which leads to elevated levels of muscle activation and an increased ability to isolate posture and BP related muscle contractions. It is known that visual cues affect sway responses^78^.

#### HDBR cardiovascular assessment

During HDBR, participants were wheeled to the test room in their beds where they were instrumented for BP, ECG, and EMG. Following instrumentation, 10 min of continuous data were recorded.

### Data collection

ECG was acquired using bipolar 3 lead ECG (FD-13, Fukuda Denshi Co. Ltd, Tokyo, Japan) in a standard Lead II electrode configuration. Continuous BP was monitored through a non-invasive Portapres (FMS, Amsterdam, The Netherlands) and the absolute blood pressure was height-corrected to heart level. Transdermal differential recording of surface EMG was performed using the Bagnoli-8 (Delsys Inc, MA, USA) EMG system from four bilateral lower leg muscles: tibialis anterior, lateral soleus, and medial and lateral gastrocnemius. The sites for EMG sensor placement were chosen based on recommendations from the SENIAM project^79^. Postural sway data, in the form of center of pressure (COP) coordinates (medial–lateral sway COPx and antero-posterior sway COPy) were derived from force and moment data obtained with an Accusway Plus force platform (AMTI, MA, USA). NIRS was recorded by a laser tissue blood oxygenation monitor (Omegawave BOM-L1 W). Data were acquired at a sampling rate of 1,000 Hz through a National Instruments USB-6218 16-bit data acquisition platform and Labview 2013 software (National Instruments Inc, TX, USA).

Calf circumference was measured using a flexible tape measure around the maximal circumference of the right and left calf with the knee bent at 90 degrees and the lower leg perpendicular to the floor. The medial calf skinfold thickness was acquired with a skinfold caliper (Meikosha, Tokyo, Japan).

### Data analysis

Data from the last five minutes of the quiet stance phase were used for analysis. Data analysis has been described in detail by Xu et al.^20^ and are provided in the supplementary materials for this paper. In brief, Morlet wavelet was applied to obtain time–frequency distributions of WTC^17,18^ for signal pair SBP → EMG_imp_ (muscle-pump baroreflex). The threshold of significant coherence was obtained through the Monte Carlo method^20^. Three frequency bands: very low frequency (VLF, 0.03–0.07 Hz), low frequency (LF, 0.07–0.15 Hz), and high frequency (HF, 0.15–0.5 Hz) were investigated in this study. The fraction of time each reflex was active (Fraction Time Active: FTA) was computed as the area above significant coherence threshold in each frequency band divided by the total area of that frequency band. The response gain value was calculated from the cross wavelet transform of the two signals^80^ and averaged over regions of significant WTC within each frequency band. A new term ‘Active’ Gain which is the product of the two values (Gain × FTA) was further used to describe the effectiveness of each reflex. The causal relationship between the signal pair EMG_imp_ ↔ SBP, was calculated using CCM method^81^. Details of the methodology can be found in Verma et al.^19^ and the supplementary material of Sugihara et al.^81^ A two-dimensional plot (Active Gain vs Causality) was used to provide a view of the relationship between causality and activity as it pertained to the muscle-pump baroreflex and HDBR. Blood pressure variability (BPV) was derived from the power spectral analysis of the SBP series from HF (0.15–0.5 Hz) and LF (0.05–0.15 Hz) bands via Welch’s method^82^ using Hanning window and 50% overlap. In this study, the square root was used as a normalizing function for spectral power. This gave the amplitude of the variations in BPV in the respective frequency bands (A_HF_, A_LF_). Mean values for all except NIRS data were averaged over the beat-to-beat sequence of the final 5 min of data collected. NIRS data were calculated as the difference between the mean 30 s value at the end of stand and the mean 30 s value at the beginning of stand.

#### Statistical analysis

Statistical significance for the data collected during standing were analyzed for the main effects of Cocktail, Bedrest and Test-days using a three-way repeated measures ANOVA (JMP14, SAS Institute). As the cocktail intervention was randomized amongst the participants, subjects were nested within Cocktail as either test or control. Given the 60-day interval between pre- and post-bedrest, to determine the effect of bedrest on the measures, test-days were nested within Bedrest (Pre-bedrest contained BDC12 and BDC02 and post-bedrest contained R0 and R8). Significance of interactions were assessed with Tukey’s HSD (post hoc) test.

Supine data were measured at multiple times during pre-, inter- and post-bedrest. These data analyzed were then analyzed for the effects of Cocktail over Test-days using a two-way with similar nesting of participants within Cocktail as outlined above. Significance over levels or interactions was assessed with Tukey’s HSD (post hoc) test. If no overall significant interaction were found between Cocktail and Test-Day, the participants were merged and a one-way repeated measures ANOVA was performed on the collapsed data set.

## Supplementary information

Supplementary information.
