# Supplementary material for: Cardio-postural interactions and muscle-pump baroreflex are severely impacted by 60-day bedrest immobilization
Source: Sci Rep. 2020 Jul 21;10:12042. doi: 10.1038/s41598-020-68962-8 (PMC7374578; doi:10.1038/s41598-020-68962-8)
Supplement: Supplementary file 1 — Supplementary information. [file 41598_2020_68962_MOESM1_ESM.docx]

**Cardio-postural interactions and muscle-pump baroreflex are severely impacted by 60-day Bedrest Immobilization**

Da Xu^1^, Malcom F. Tremblay^1^, Ajay K. Verma^2^, Kouhyar Tavakolian^2^, Nandu Goswami^3^, Andrew P. Blaber^1^

^1^Department of Biomedical Physiology and Kinesiology, Simon Fraser University, Burnaby- V5A 1S6, Canada.

^2^School of Electrical Engineering and Computer Science, University of North Dakota, Grand Forks-58202, USA.

^3^Physiology Division, Otto Loewi Research Center for Vascular Biology, Immunology and Inflammation, Medical University of Graz, Austria.

SUPPLEMENTAL MATERIAL

**Supplemental Methods**

Selection Criteria

*Inclusion criteria* were selected to ensure participant safety and quality of data. Male healthy volunteers between 20 and 45 years old, 158-190 cm tall, and BMI between 22 and 27 kg/m^2^ were selected. Fitness level was controlled by V̇O_2_max assessment: for participants < 35 years, 35 mL/kg/min. < V̇O_2_max < 60 mL/kg/min., for participants > 35 years, 30 mL/kg/min. < V̇O_2_max < 60 mL/kg/min. Volunteers were only considered if they were non-smokers and had no alcohol or drug dependency.

*Exclusion criteria* were chosen to ensure participant safety during bedrest and eliminate confounding factors to the individual protocols being carried out. Volunteers were excluded if they presented with history of orthostatic intolerance, cardiac rhythm disorders, chronic back pain, history of hiatus hernia or gastro-esophageal reflux, abnormal results from lower limb echo-Doppler, or personal or familial history of thrombophlebitis, thrombosis, or positive response to thrombosis screening procedure. History of thyroid dysfunction, renal stones, diabetes, migraines, genetic muscle and bone disease, and sleep disorders were grounds for exclusion. Bone mineral density T-score ≤ -1.5, osteosynthesis material, presence of metallic implants, and a history of knee problems, joint surgery, or broken legs were grounds for exclusion due to the known effect of bedrest on bone density and demineralization. Sensory considerations resulting in exclusion included vestibular disorders, audition problems, vision corrected to no more than 20/30, color blindness, or active or a history of claustrophobia. Exclusion on the basis of diet restrictions, including but not limited to vegetarian or vegan preferences and allergies to peanut or soya, was enforced due to the strict caloric and macronutrient control in the supplied diet. Biological samples prevented participants from having allergy to xylocaine, poor tolerance to blood sampling, having given more than 8mL/kg of blood in the 8 weeks leading up to the study, or positive reaction to the following tests: HVA IgM (hepatitis A), HBs antigen (hepatitis B), anti-HVC antibodies (hepatitis C), anti-HIV1+2 antibodies. Participants were excluded if they were already participating in or in the exclusion period of another study, had received more than 4500 Euros for being a research participant within the last 12 months, were incarcerated, were under guardianship or trusteeship, or refused to grant permission to contact their general practitioner. The volunteers also could not be engaged in shift work or travel across more than one-time zone within 2 months prior to the study. Finally, volunteers who, in the judgment of the investigator, were likely to be non-compliant during the study or unable to cooperate because of a language problem or poor mental development were also excluded.

Rules for behaviour during bedrest

The following instruction were given to all the bed rest participants as standard procedures and requirements and included in the ethics documents. Similar procedures have been in use by the European Space Agency for previous bed rest studies [1].

Participants were to follow a day-night cycle of 7 am wake-up and lights-out at 11 pm. Participants were instructed that during bed rest they were allowed to change position in bed, but that physical activity was to be kept at a minimum level and all hygiene needs were to be conducted in the head-down tilt position. Showering was performed in a dedicated head-down tilt shower-bed. Participants were permitted to lie on their stomach, back or side, however, they were instructed that, when ~~in~~ lying on their side the trunk and head needed to remain in the head-down position (i.e. the head could not be held up with the hand/arm as one commonly does when reading). Video supervision on a 24-hour basis was conducted to permit monitoring of participant adherence to the study protocol.

Naps were not allowed, and they were not allowed to lie on their beds during the day during the baseline data collection period (BDC).

The participants were to finish their tray meals.

They are not allowed to leave the facility, except for some specific measures (MRI for example) and in that case they were always with somebody of the staff.

They were to respect the schedule of the study and of the scientific tests. They were to respect the instructions given by the staff and by the scientists during the tests.

They were allowed phone calls, but these were not to interfere with the schedule of the scientific tests or the other activities.

Nutritional Supplementation

Half of the participants received daily capsule supplements of antioxidants, vitamin E-selenium, and omega-3. The antioxidant cocktail (XXS-2A-BR2 formulation, produced by the company Spiral) was composed of 6 bioactive polyphenols extracted from food plants. Administered by 6 daily capsules (2 capsules at each meal), the daily dose was approximately 741mg. A commercially available vitamin E-selenium capsule produced by Solgar was administered daily with breakfast, giving daily doses of 168mg of vitamin E and 80µg of selenium. One omega-3-acid ethyl ester capsule containing EPA ethyl ester and DHA ethyl ester, produced by Omacor®, was taken with every meal. The daily dose of EPA was 1.1g and DHA was 1g. Nutrition was strictly controlled. There was a set meal plan with a rotating menu designed to ensure exact intake of calories and macronutrients. Caffeine and alcohol were strictly prohibited for the entire study. Participants were compelled to finish each meal, and portions sizes were calculated relative to each participant’s body mass on their first morning of the ambulatory period before bed rest (**Supplemental Table**).

Data Analysis

Data from the last five minutes of the quiet stance phase were used for analysis. Data analysis was described in detail by Xu et al..[1] The QRS complex was detected from electrocardiogram (ECG) to form the time series of heart beat period (i.e., RR-interval) and heart rate (HR). Systolic blood pressure (SBP) was obtained from the continuous BP within each RR-interval. Aggregate electromyography (EMG) was obtained by addition of rectified EMG signals from all individual leg muscles to represent the overall muscle activities. The EMG envelope was then captured by a moving average filter. Finally, analogous to the impulse of force, the EMG impulse (EMG_imp_) was calculated as the area under the aggregate EMG envelope within each heartbeat to represent the muscle contraction strength on a beat-by-beat basis. The concept of impulse was employed because, in a beat-by-beat perspective, the strength of muscle contraction over a heartbeat would be related to the length of that beat. That is, a brief strong contraction can be considered to be equivalent to weaker contractions over a longer period and the same contraction level would produce higher overall strength over a longer heartbeat. The resultant centre of pressure (COPr) was COPy (i.e., $\text{COPr}\text{=}\sqrt{\text{COPx}^{\text{2}}\text{+}\text{COPy}^{\text{2}}}$ $\text{COPr=}\sqrt{\text{COPx}^{\text{2}}\text{+}\text{COPy}^{\text{2}}}$) and the change rate of COPr (COPr_v_) was calculated as the first derivative of COPr and averaged within each beat. All beat-by-beat time series were resampled to 10 Hz using spline interpolation prior to the wavelet transform and causality analysis.

*Wavelet transform coherence*: The Morlet wavelet was applied to obtain time-frequency distributions of wavelet transform coherence (WTC)[3,4] for signal pair SBP→EMG_imp_ (muscle-pump baroreflex). The threshold of significant coherence was obtained through the Monte Carlo method.[2] Three frequency bands: very low frequency (VLF, 0.03 – 0.07 Hz), low frequency (LF, 0.07 – 0.15 Hz), and high frequency (HF, 0.15 – 0.5 Hz) were investigated in this study. The three frequency bands were divided into 49 sub-band segments (15 in VLF, 13 in LF, and 21 in HF).[2] The fraction time active (FTA) was computed as the area above significant coherence threshold in each frequency band divided by the total area of that frequency band. The response gain value was calculated from the cross wavelet transform of the two signals[5] and averaged over regions of significant WTC within each frequency band.

*Cause-and-effect relationship*: Causal relationship between signal pairs of interest (EMG_imp_↔SBP,) was calculated using convergent cross mapping (CCM) method.[6] The strength of causal direction in the forward and reverse direction was quantified by calculating correlation coefficient between the original signal and its estimate.[7] The manifold of each signal was constructed using the time delay and embedding dimension. For cardio-postural signals, the optimal dimension of reconstruction to capture physiological alterations within a heartbeat range was determined to be 4 based on the minimization of false nearest neighbor at a delay of 10 samples. Details of the methodology can be found in Verma et al.[7] and the supplementary material of Sugihara et al.[6]

*Gain, FTA and Causality Relationship*: We chose to explore the interrelationship between gain, FTA and causality to develop an index of reflex efficiency. With the observed changes in both gain and FTA, it could be expected that a true estimate of the effectiveness of the system would be a function of both. The application of the interaction can only occur when the two systems are interacting which is reflected in the determination of the fraction time active. That is, the operating gain is only effective during the time of active interaction. We therefore developed a new term ‘Active’ Gain which is the product of the two values (Gain×FTA). Causality (between 0 and 1) is an estimate of strength of the directionality between the signals, with lower value indicating a weaker causal relationship. A two-dimensional plot (Active Gain vs Causality) was developed to provide a view of this interaction as it pertained to the muscle-pump baroreflex in relation to HDBR.

References

1. Belavý DL, Bock O, Börst H, Armbrecht G, Gast U, Degner C, Beller G, Soll H, Salanova M, Habazettl H, Heer M, de Haan A, Stegeman DF, Cerretelli P, Blottner D, Rittweger J, Gelfi C, Kornak U, Felsenberg D. The 2^nd^ Berlin bedRest Sstudy: protocol and implementation. *J Musculoskelet Neuronal Interact* 2010;10(3):207-219.

2. Xu D, Verma AK, Garg A, Bruner M, Fazel-Rezai R, Blaber AP, Tavakolian K. Significant role of the cardiopostural interaction in blood pressure regulation during standing. *Am J Physiol Heart Circ Physiol*. 2017;313:H568–H577.

3. Garg A, Xu D, Blaber AP. Statistical validation of wavelet transform coherence method to assess the transfer of calf muscle activation to blood pressure during quiet standing. *Biomed Eng Online*. 2013;12:132.

4. Garg A, Xu D, Laurin A, Blaber AP. Physiological interdependence of the cardiovascular and postural control systems under orthostatic stress. *Am J Physiol Heart Circ Physiol*. 2014;307:H259-64.

5. Grinsted A, Moore JC, Jevrejeva S. Application of the cross wavelet transform and wavelet coherence to geophysical time series. *Nonlinear Process Geophys*. 2004;11:561–566.

6. Sugihara G, May R, Ye H, Hsieh C, Deyle E, Fogarty M, Munch S. Detecting causality in complex ecosystems. *Science*. 2012;338:496–500.

7. Verma AK, Garg A, Xu D, Bruner M, Fazel-Rezai R, Blaber AP, Tavakolian K. Skeletal muscle pump drives control of cardiovascular and postural systems. *Sci Rep*. 2017;7:45301.

| **Supplemental Table:** Calculations of caloric intake (macronutrients, micronutrients) | |
| --- | --- |
| **Dietary Factor** | **Dose** |
| Liquid Intake | between 35 and 50 mL/kg/day |
| Caloric Intake | Age<30y: BMR=15.3 x body weight (kg) + 679  Age>30y: BMR=11.6 x body weight (kg) + 879 |
| Proteins | 1-1.2 g/kg(body weight)/day |
| Fat | 30-35% TEE |
| Carbohydrates | Remaining TEE |
| Sodium | 3000 - 4500 mg/day |
| Potassium | 3500 - 5000 mg/day |
| Calcium | 1000-1200 mg/day |
| Chloride | 5500 - 7500 mg/day |
| BMR: basal metabolic rate, TEE: total energy expenditure | |
